# A survey to evaluate parameters governing the selection and application of extracellular vesicle isolation methods

**DOI:** 10.1177/20417314231155114

**Published:** 2023-03-08

**Authors:** Soraya Williams, Aveen R Jalal, Mark P Lewis, Owen G Davies

**Affiliations:** School of Sport, Exercise and Health Sciences, Loughborough University, Loughborough, UK

**Keywords:** Extracellular vesicles, isolation methods, therapeutics, application, survey

## Abstract

Extracellular vesicles (EVs) continue to gain interest across the scientific community for diagnostic and therapeutic applications. As EV applications diversify, it is essential that researchers are aware of challenges, in particular the compatibility of EV isolation methods with downstream applications and their clinical translation. We report outcomes of the first cross-comparison study looking to determine parameters (EV source, starting volume, operator experience, application and implementation parameters such as cost and scalability) governing the selection of popular EV isolation methods across disciplines. Our findings highlighted an increased clinical focus, with 36% of respondents applying EVs in therapeutics and diagnostics. Data indicated preferential selection of ultracentrifugation for therapeutic applications, precipitation reagents in clinical settings and size exclusion chromatography for diagnostic applications utilising biofluids. Method selection was influenced by operator experience, with increased method diversity when EV research was not the respondents primary focus. Application and implementation criteria were indicated to be major influencers in method selection, with UC and SEC chosen for their abilities to process large and small volumes, respectively. Overall, we identified parameters influencing method selection across the breadth of EV science, providing a valuable overview of practical considerations for the effective translation of research outcomes.

## Introduction

Extracellular Vesicles (EVs) play an important role in a wide range of physiological and pathophysiological processes.^1^ These findings have resulted in an increasing number of studies isolating EVs from a range of biofluids and cell sources for diverse applications in diagnostics and therapeutics. Growing interest in the field is exemplified by the number of EV publications doubling from 3000 to 6000 per year in the last 5 years.^2^ The presence of EVs in biofluids such as urine,^3^ blood plasma,^4^ serum^5^ and saliva,^6^ just to name a few, make EVs prospective biomarker candidates that can be readily obtained utilising non-invasive processes for applications in diagnostics.^7^ Their broad functional roles in providing a transport network for the exchange of bioactive cargos between cells and tissues of the body also provides a framework for the development of EV therapeutics. EVs are appealing therapeutic candidates due to their inherent biocompatibility, small size and ability to cross complex tissue and cell barriers. This has led to pioneering studies demonstrating the effective use of EVs to deliver therapeutics such as siRNAs^8^ to challenging targets such as the brain (e.g. across the blood-brain barrier).^9,10^ The therapeutic capacity of EVs has also been documented pre-clinically for applications in wound healing,^11^ diabetes,^12^ and as drug delivery vehicles.^13–15^ When compared to cell-based therapies, EVs present a comparatively safe alternative due to the fact they do not replicate and have a low risk of inducing an immunogenic response.^16,17^ Lastly, EVs are commonly stored at −80°C, with alternative storage methods such a lyophilisation gaining interest and increasing the feasibility of delivering an off-the-shelf therapeutic in the future.^18,19^ All of the above have led to a growth in EV publications across a range of disciplines from cell biology^20,21^ to materials science^22–24^ and bioengineering.^25,26^ For a comprehensive overview of the emerging therapeutic applications of EVs we recommend the article by Nagelkerke et al.^27^

Whilst an increased application of EVs across disciplines is advantageous to the progression of novel therapeutics and diagnostics, it is widely acknowledged that current EV isolation methods can result in varying outputs in terms of yield, purity and reproducibility.^28^ These variations can impact downstream biological functions and thus the utility of outputs for applications such as therapeutics and diagnostics. As such, it is essential that all researchers applying EVs in their studies are aware of current limitations within the field and how this relates to the purity, scalability and the compatibility of isolation methods with intended downstream applications. The absence of a gold-standard EV isolation method was first highlighted in the minimal information for studies of extracellular vesicles (MISEV), published by the International Society for Extracellular Vesicles (ISEV) in 2015^29^ and updated in 2018.^30^ These publications provided the first framework of experimental guidelines on EV isolation and analysis based on a continually evolving collective knowledge. To gauge employment of isolation methods within the EV field, the ISEV Rigor and Standardization Subcommittee distributed an international survey in 2015 looking at ‘techniques used for the isolation and characterization of extracellular vesicles’.^31^ This was followed up with a second survey in 2019^32^ that provided an update on trends and developments for EV isolation and analysis, in order to identify evolving challenges. The 2015 survey^31^ provided an overview of trends for fundamental parameters such as primary isolation method, EV source, starting volume, characterisation and downstream analysis (e.g. in vitro functional analyses, RNA analysis, proteomic analysis, flow cytometry, in vivo functional analyses and lipidomics). The follow-up survey in 2019^32^ provided more of a focus on quality control but highlighted increased diversity of EV sources and isolation methods applied, as well as increased usage of characterisation methods to validate the presence of EVs by respondents (4–6 EV characterisation methods applied in 2019 compared to ⩽3 in 2015). The overall increased diversity and application of isolation and analysis methods between 2015 and 2019, highlighted the rate at which the field is expanding. As the EV field continues to expand across disciplines and prospective EV therapies begin to emerge, it is of increasing importance that we understand how EV isolation methods are being applied and define both scientific and pragmatic considerations surrounding method selection for varying disciplines and research environments.

In this publication we report the findings of our survey, providing a broad overview of up-to-date trends in the utilisation of EV isolation methods and parameters that govern their selection, in addition to an overview of the breadth of application of EVs and diversity of disciplines conducting EV studies. These findings enabled the very first cross-comparison study across disciplines and sectors to provide a comprehensive overview of the selection and application of EV isolation methods and the parameters governing their implementation. Ultimately, these factors will influence the rate and efficiency for translation of prospective EV therapeutics both commercially and clinically. A summary of the methodology, as well as the advantages and limitations of the EV isolation methods discussed in this study can be found in Table 1.

**Table 1.** Summary of EV isolation methodology discussed in this study and their advantages and limitations.

Isolation Method	Methodology	Advantages	Limitations
Ultracentrifugation (UC)	Utilises a series of high spin speeds (e.g. 10,000×*g* 30 min followed by 120,000×*g* for 70 min) to pellet EVs	Cost efficient, does not require additional reagents, ability to process relatively large sample volumes in a laboratory setting	Requires specialist equipment, low purity outputs, limited reproducibility, limited scalability with industrial volumes, EV recovery impacted by rotor type and g-force applied
Size exclusion chromatography (SEC)	Separation of EVs by size into several fractions when passing through a gel column containing beads with known pore sizes	Cost efficient (dependent on whether columns are purchased commercially or made in-house), enhanced purity; in particular when isolating from biofluids	Often requires additional concentration steps for large sample volumes (e.g. ultrafiltration) impacting EV recovery and resulting in increased costs, requires specialist equipment
Polyethylene (PEG) Precipitation	EV source incubated with PEG solution to precipitate EVs which are then pelleted using conventional centrifugation	Cost-efficient, scalable, simple methodology not requiring specialist equipment	Co-precipitation of proteins, residual presence of polymer interferes with downstream analysis and has potential clinical safety concerns
Commercial Reagents (e.g. Total Exosome Isolation Reagent and ExoQuick)	EV source incubated with commercial reagent to precipitate EVs which are then pelleted using either UC or conventional centrifugation	Simple and easily accessible methodology	Co-precipitation of proteins, residual presence of polymer interferes with downstream analysis and has potential clinical safety concerns, costly when isolating from large volumes, reliant on continued availability of product
Tangential flow filtration (TFF)	Crossflow filtration where the mainstream flows parallel to the membrane face allowing a continuous cycle with applied pressure	Scalable, batch-to-batch consistency	Reduced purity with high protein samples due to membrane fowling, requires specialist equipment
Microfluidics	Processing of EV sources in solution using microchannels	Rapid processing, selectivity, enhanced purity	Cost, small sample processing, device complexity
Immunoaffinity techniques	Capture of EVs based on specific surface markers	Selectivity, enhanced purity	Cost, requires knowledge of EV markers, limited scalability
Aqueous-two phase system	Samples incubated with PEG and DEX in solution and centrifuged to sperate EVs into the bottom DEX phase and proteins into the top PEG phase	Cost-efficient, simple methodology not requiring specialist equipment, scalable, potentially enhanced purification when compared to standard precipitation methods	EVs are recovered in DEX a viscous reagent which can interfere with downstream analysis and has unknown clinical safety

## Survey

This survey was generated to observe current trends in EV isolation methods and the parameters that impact their application. These influencing parameters included research setting, experience, EV source, starting volume, primary research focus, application (i.e. therapeutics and diagnostics) and implementation factors (i.e. equipment availability, cost and time efficiency). The full survey can be found in the Supplemental Materials. The survey was opened November 2020 and closed February 2021. It was distributed via a mailing list containing ISEV and UK Society of EVs (UKEV) affiliated PIs and members, as well as industry partners such as NanoFCM Co., Ltd, (Nottingham). Alongside this, the survey was posted on social media platforms such as Twitter and LinkedIn, where it was supported and shared by EV groups, including UKEV and the international student network on EVs (SNEV). Information on the survey was also disseminated at the 2020 December UKEV annual meeting. All data was collated, analysed and graphical outputs produced using Qualtrics survey software. A total of 87 complete responses were utilised to generate the data in this study. The survey was approved by Loughborough University ethics committee (Reference 2020-2478-2026). Respondents remained anonymous with informed consent obtained prior to participation (details in Supplemental Materials). The following inclusion criteria was required for participation: participants current research must involve EVs, they must have had personal experimental experience of EV isolation methods and questions must be answered based on these personal practical experiences.

## Research setting and experience

**Figure 1.**
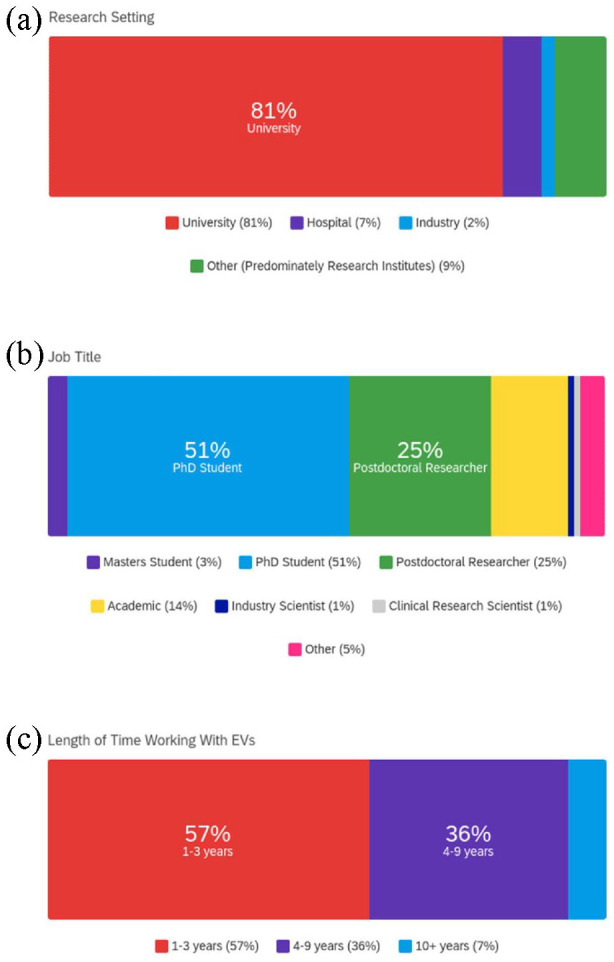
Respondents research setting and experience: (a) research setting (where other is selected research settings included research institutes and government), (b) job title and (c) length of time working with EVs.

Of the 87 respondents 81% were from an academic background, 7% from a hospital setting, 2% from an industry setting and 9% selected *other*, which consisted of research institutes and government (Figure 1(a)). The data collected also indicated that respondents had a range of job titles which included MSc students (3%), PhD students (51%), postdoctoral researchers (25%), academics (14%), industry scientists (1%), clinical research scientists (1%) and *other* (5%) which included managing director and clinician scientist (Figure 1(b)). In addition to research setting and job title, we evaluated how long respondents had worked with EVs. The majority (over half, 57%) of respondents had worked with EVs for 1–3 years, 36% 4–9 years and 7% 10+ years (Figure 1(c)). These outcomes demonstrated that respondents had a range of experience, in terms of both career stage and working with EVs across varied research settings. Out of these respondents 78% said that EV research was their primary area of focus.

## Application and source of EVs

**Figure 2.**
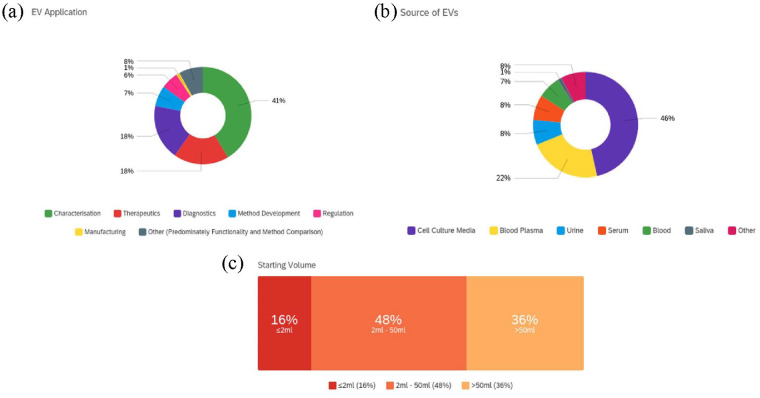
EV Source, starting volume and application of EVs: (a) primary application of EVs, (b) source of EVs, all applicable were selected (where other was selected EV sources included ascites, parasite culture media, amniotic fluid, milk, zebra fish, synovial fluid, bacterial growth media, tissue, semen, cerebrospinal fluid, plural fluid and bronchoalveolar lavage fluid) and (c) starting volume.

When assessing the primary application of respondents, the data indicated a principal focus on EV characterisation (41%), followed by therapeutics and diagnostics (18%), method development (7%), regulation (6%), manufacturing (1%) and *other* (8%). Where *other* was selected, applications included functionality studies, mechanistic understanding, prognostics, comparison studies and communication between hosts and pathogens (Figure 2(a)). When evaluating EV sources used (multiple answer selection where applicable) data showed the majority of respondents isolated EVs from cell culture media (46%), followed by blood plasma (22%), urine (8%), serum (8%), blood (7%), saliva (1%) and *other* (8%). Where *other* was selected, EV sources consisted of ascites, parasite culture media, amniotic fluid, milk, zebra fish, synovial fluid, bacterial growth media, tissue, semen, cerebrospinal fluid, plural fluid and bronchoalveolar lavage fluid (Figure 2(b)). The data also informed of respondents starting volume, with 16% utilising a volume of ⩽2 mL, 48% between 2 and 5 mL and 36% >50 mL (Figure 2(c)).

## EV Isolation methods and parameters governing method selection

**Figure 3.**
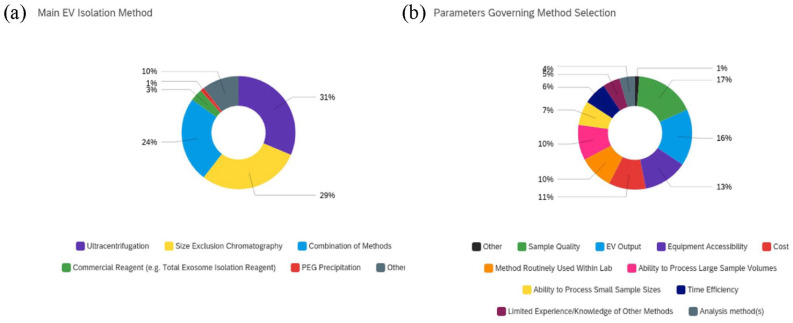
EV Isolation methods and parameters governing method selection: (a) main EV isolation method (where other is selected EV isolation methods included tangential flow filtration, density gradient, ultrafiltration and microfluidics) and (b) parameters governing method selection, all applicable were selected.

When respondents selected their main EV isolation method we saw that ultracentrifugation (UC, 31%), size exclusion chromatography (SEC, 29%) and a combination of methods (24%) were most applied. Only 3% of participants used commercial reagents, 1% polyethylene glycol (PEG) precipitation and 10% *other*. Where *other* was selected, EV isolation methods included tangential flow filtration (TFF), density gradient (DG), ultrafiltration (UF) and microfluidics (MFs) (Figure 3(a)). When selecting parameters governing this method selection (multiple answer selection), the results showed that several factors played a role, with no one major limiting factor highlighted. The two influencing factors most selected were sample quality (17%) and EV output (16%). These were subsequently followed by equipment accessibility (13%), cost (11%), adoption of methods routinely applied within the respondents lab (10%) and ability to process large sample volumes (10%). The factors which least impacted isolation method selection were post-isolation analysis methods (4%), self-identified limited experience/knowledge of other methods (5%), time efficiency (6%) and ability to process small sample volumes (7%) (Figure 3(b)).

## Isolation method based on research setting and experience

**Figure 4.**
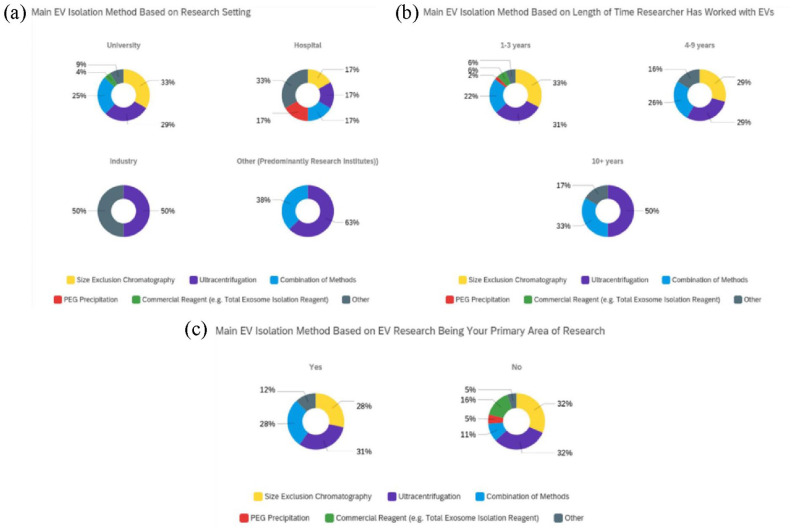
Responses classified by their main EV isolation method based on research setting and experience: (a) research setting, (b) EV research experience and (c) primary research focus on EVs. EV isolation methods include ultracentrifugation, size exclusion chromatography, a combination of methods, PEG precipitation and commercial reagents.

When cross-comparing the main EV isolation method utilised and research setting (Figure 4(a)), the methods most applied in universities (81% of total respondents, Figure 1(a)) were SEC (33%), UC (29%) and a combination of methods (25%). In hospitals (7%, of total respondents, Figure 1(a)), a greater variety of methods were employed, with an increased application of PEG precipitation (17%) and *other* methods (33%, included UF). In addition to a decrease in the use of SEC (17%), UC (17%) and a combination of methods (17%). Respondents working in industry (2%, of total respondents, Figure 1(a)) utilised an equal split (50%) of UC and *other* methods (included TFF). However, data from industry only accounted for 2% of total respondents (Figure 1(a)) which should be recognised when interpreting the data. Those working in *other* research settings (9%, research institutes and government, Figure 1(a)) applied UC (63%) and a combination of methods (38%).

When cross referencing data generated on method selection with research experience (Figure 4(b)), UC and a combination of methods were found to be most commonly applied, irrespective of experience. Those working with EVs for 1–3 years (57% of total respondents, Figure 1(c)) most frequently applied SEC (33%), UC (31%) and a combination of methods (22%). Respondents of this level of experience also showed utilisation of a greater variety of methods, with commercial reagents (6%) and PEG precipitation (2%) more prevalent compared to respondents who had worked with EVs for over 3 years. Respondents working with EVs for 4–9 years (36%, of total respondents, Figure 1(c)) displayed an increased use of *other* methods (16%, included TFF), as well as the use of common methods such as SEC (29%), UC (29%) and a combination of methods (26%). Those with 10+ years experience (7%, of total respondents, Figure 1(c)) showed similar use of *other* methods (17%, included TFF) and a combination of methods (33%) to those working with EVs for 4–9 years. As well as increased use of UC (50%) and no recorded use of SEC.

Lastly, we cross compared data to determine whether selection of an EV isolation method was influenced by respondents identifying EV research as their primary focus (Figure 4(c)). Data indicated that UC and SEC remained popular methods (28%–32%) amongst respondents with (78%, of total respondents) and without (22%, of total respondents) a primary research focus on EVs. Where EV research was not the primary research focus there was additional use of PEG precipitation (5%) and commercial reagents (16%), as well as decreased usage of a combination of methods (11%) and *other* methods (5%).

## Isolation method based on EV source and starting volume

**Figure 5.**
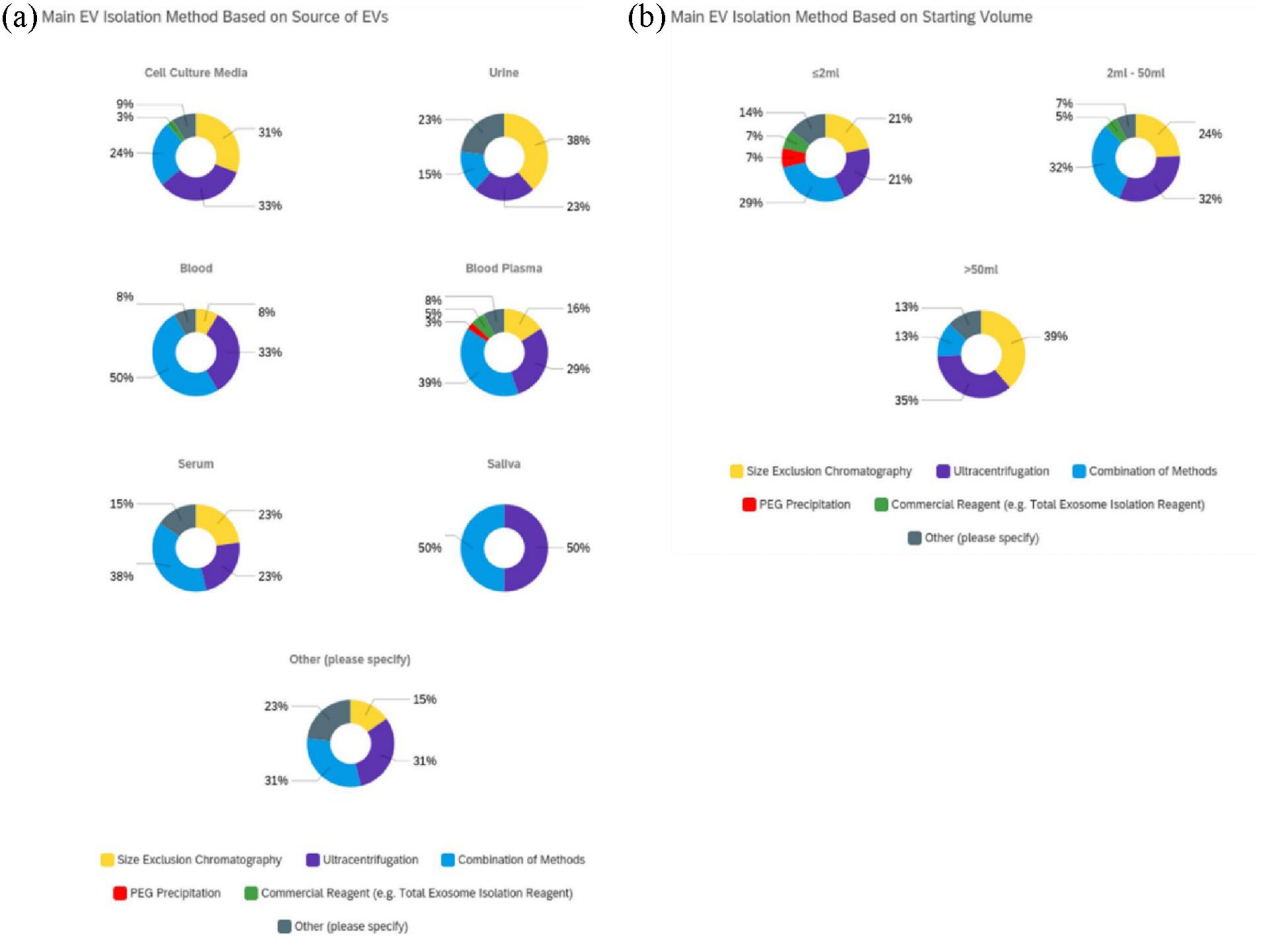
Respondents main EV isolation method based on EV source and starting volume: (a) source of EVs and (b) starting volume. EV isolation methods include ultracentrifugation, size exclusion chromatography, a combination of methods, PEG precipitation and commercial reagents.

When observing the impact of EV source on isolation method selection (Figure 5(a)), data generated showed that SEC, UC and a combination of methods were utilised for all EV sources recorded (method usage varied by source). When isolating from urine (8%, of total respondents, Figure 2(b)) there was increased use of SEC (38%) and *other* methods (23%, included UF, DG and TFF). When isolating EVs from blood (7%, of total respondents, Figure 2(b)) there was increased use of a combination of methods (50%) and for blood plasma (22%, of total respondents, Figure 2(b)) the additional usage of PEG precipitation (3%) and increased use of commercial reagents (5%). For saliva (1%, of total respondents, Figure 2(b)) the results showed UC or a combination of methods (50% split) was preferred. However, it should be acknowledged that saliva as a source of EVs accounted for just 1% of respondents (Figure 2(b)).

Data was also cross-compared to determine method selection based on starting volume (Figure 5(b)). Outcomes indicated that when isolating EVs from a volume of ⩽2 mL (16%, of total respondents, Figure 2(c)), that there was greater variety in the methods applied. UC (21%), SEC (21%) and a combination of methods (29%) were indicated to be most frequently selected for use with these small sample volumes. In addition to PEG precipitation (7%) and increased utilisation of commercial reagents (7%). For a starting volume of 2–50 mL (48%, of total respondents, Figure 2(c)) it was observed that SEC (24%), UC (32%) and a combination of methods (32%) remained most utilised, with no evidence of the application of PEG precipitation. The use of PEG precipitation was also not recorded when isolating from a volume >50 mL (36%, of total respondents, Figure 2(c)). This was also true for commercial reagents. Data indicated that for these larger volume samples of >50 mL there was an increased use of SEC (39%) and decreased use of a combination of methods (13%).

## Isolation method based on application and implementation

**Figure 6.**
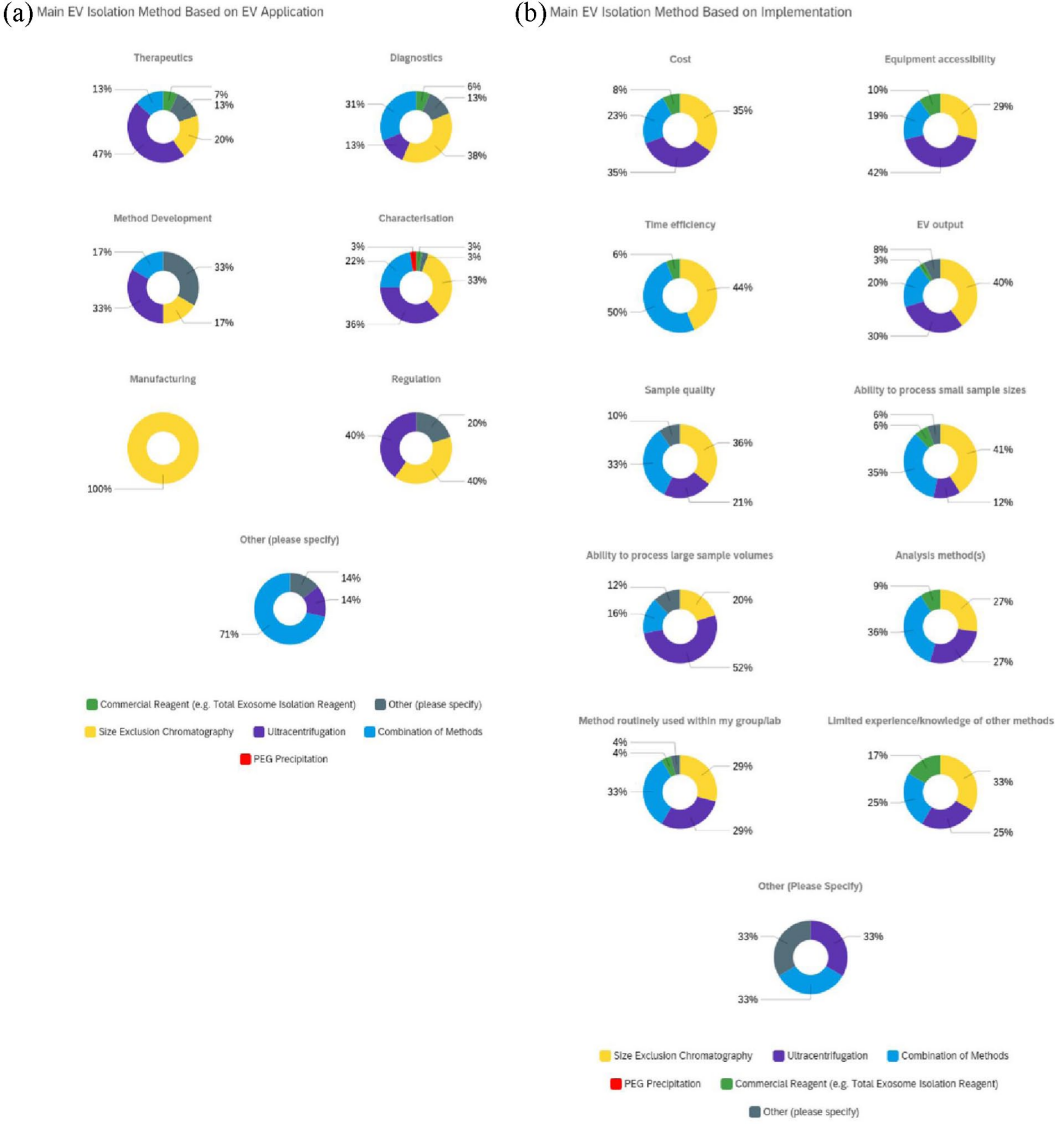
Main EV isolation method selection based on application and implementation: (a) application and (b) implementation. EV isolation methods include ultracentrifugation, size exclusion chromatography, a combination of methods, PEG precipitation and commercial reagents.

When looking at the impact of application on method selection (Figure 6(a)), data indicated that for therapeutics (18%, of total respondents, Figure 2(a)) UC (47%) was the predominant method of isolation, followed by SEC (20%). However, for diagnostics (18%, of total respondents, Figure 2(a)) the use of UC (13%) was reduced and the use of SEC (38%) and a combination of methods (31%) increased. For research on method development (7%, of total respondents, Figure 2(a)) UC (33%) and *other* methods (33%, included MFs) were most applied. Respondents focussing on EV characterisation (41%, of total respondents, Figure 2(a)) applied a greater variety of methods, with the most popular methods utilised including UC (36%) and SEC (33%). The data generated also indicated that those working in manufacturing only reported the use of SEC. However, this accounted for just 1% of respondents (Figure 2(a)) and should be noted when interpreting the data. Those respondents working on EV regulation (6%, of total respondents, Figure 2(a)) primarily applied UC (40%) and SEC (40%) and displayed no recorded use of a combination of methods. When selecting *other* applications (8%, of total respondents, Figure 2(a)), which included mechanistic understanding, prognostics, comparison studies and communication between hosts and pathogens, an increased use of a combination of methods was recorded (71%).

Upon evaluating implementation parameters integral to method selection (Figure 6(b)), data showed that SEC (35%) and UC (35%) were regarded as the most cost-efficient. UC was also the preferred method when considering equipment accessibility (42%) and the ability to process large sample volumes (52%). In contrast, for time efficiency UC was not considered optimal (no recorded responses), whilst SEC (44%) and a combination of methods (50%) were frequently selected. SEC (41%) and a combination of methods (35%) were also most widely applied to process small sample sizes. When selecting methods based on post-isolation parameters, data indicated that for EV output SEC (40%) was the preferred method. When considering down-stream analysis, a combination of methods (36%) was preferentially selected. In addition, for sample quality both SEC (36%) and a combination of methods (33%) were the most frequently applied. Where respondents self-identified as having limited experience/knowledge of other methods there was an increased application of commercial reagents (17%). Where *other* implementation parameters was selected, respondents stated that methods were specifically optimised for their work. This was further indicated by the increased use of *other* EV isolation methods (33%) upon selecting this response. However, this accounted for only 1% of total respondents which should be noted upon evaluation (Figure 3(b)).

## Discussion

The integral role of EVs in intercellular communication, along with their prospective advantages over cell-based therapies, such as increased safety, potential for delivery to challenging targets and their capacity for off-the shelf applications, exemplifies their clinical prospects.^16,33^ These advantages have led to increased interest across diverse disciplines including clinical biomarker discovery,^34^ bioengineering^35^ and drug delivery.^36^ However, when applying EVs across such a breadth of disciplines, it is important that we not only identify the strengths and limitations of EV isolation methods but also begin to determine potential logistical, pragmatic or discipline- and sample-specific considerations that could impact the selection of EV isolation methods and thus EV recovery and clinical translation. The lack of a one-size-fits-all approach to EV isolation has been previously emphasised in the 2018 MISEV guidelines.^30^ However, to date no study has sought to comprehensively evaluate what factors govern the selection of a given isolation method across the breadth of the EV field. This survey collated the opinions of 87 respondents to evaluate their choice of EV isolation method and determine what parameters had the greatest influence on this selection. Respondents were from a range of career stages (MSC and PhD students, postdoctoral researchers, academics, industry and clinical research scientists), with length of time working with EVs ranging from: 1 to 3 years (57%), 4 to 9 years (36%) and 10+ years (7%). In addition to working in a range of research settings (majority from an academic background, 81%) and disciplines, with (78%) and without (22%) a primary focus on EV research.

The most frequently utilised EV sources by respondents were cell culture conditioned media (46%) and blood plasma (22%). These trends both aligned with those seen in the previous 2019 ISEV survey.^32^ When respondents selected their primary downstream application, there was a focus on characterisation (41%). Our data also indicated increased applications in therapeutics (18%) and diagnostics (18%), aligning with increasing numbers of clinically focused EV studies and active clinical trials.^27,37^ Method development was also selected as an application (7% of total respondents), indicating that it still remains an area of continued growth.^38^ When observing factors governing the implementation of EV isolation methods, our data indicated that there was no one major limiting factor. It was suggested that multiple factors play a role, with the two most influential factors being sample quality (17%) and EV output (16%). Conversely, respondents selected downstream analysis (4%) as the factor with the least impact on method selection. This is an interesting outcome since incompatibility of isolation methods with downstream analyses can lead to inaccurate characterisation and the potential for inaccurate findings.^39^ For example, the residual presence of precipitation reagents in isolated EV fractions can negatively impact downstream morphological assessment such as TEM imaging^40^ and omics-based sample analysis when applying methods such as mass spectrometry.^41^ This can result in unreliable identification of biomarkers^42^ and therapeutic mechanism of actions^43^ impacting clinical translation.^44^

UC remains the most widely applied method (31%) across all research settings. This finding aligned with ISEV surveys, where from 2015 to 2019^31,32^ UC continued to be preferentially selected despite increased method diversity. Outcomes from the present study suggest that UC continues to be the most universally applied method for EV isolation irrespective of EV source and is favoured for its ability to process large sample volumes (52%). In addition, we observed a reduced application of UC for processing smaller volumes (⩽2 mL, 16% of total respondents). This highlights that isolation method selection is influenced by sample volume and was consistent with the 2015 ISEV survey findings. The scalability of UC was further indicated by its favoured selection for therapeutic applications (47%), aligning with its reported utilisation in clinical trials.^45^ It should be noted, that while UC is scalable, EV recovery is influenced by factors such as the centrifuge rotor and g-force applied.^28^ However, accessibility (47%) likely plays a role in the application of UC across all parameters.

The second most applied method overall was SEC (29%). This method was most popular when isolating EVs for diagnostics (38%) and characterisation studies (33%). Moreover, SEC was viewed to be equally as cost effective as UC (35%), highlighting its perceived potential application for larger therapeutic studies. SEC was preferentially selected for the isolation of EVs from urine (38%), while a combination of methods was selected when isolating EVs from blood (50%) and its components plasma (39%) and serum (38%). There are a number of factors which might influence the preferential selection of an isolation method for a given EV source, such as sample viscosity, lipid and protein content and source-specific contamination.^39^ For example, the presence of Tamm-Horsfall protein (THP) in urine and high-density lipoproteins (HDLs) in blood and it is components (plasma and serum) are known to aggregate and encapsulate EVs, resulting in artifacts^46,47^ that can be readily observed using TEM.^48,49^ These artefacts could also mask therapeutic/diagnostic molecules of interest when applying analysis methods such as mass spectrometry. This can often be resolved through the incorporation of reducing agents (e.g. dimethylammonio]-1-propanesulfonate)^50^ and anticoagulants.^51,52^ However, proteins attributable to disease related changes, such as the highly abundant presence of albumin with renal disease nephrotic syndrome, can also interfere with methods such as UC and UF^53,54^ and result in masking or misidentification of diagnostic markers. These challenges when working with biofluids have been highlighted by both the ISEV 2019 blood task force statement^55^ and the ISEV urine task force 2021 position paper.^53^ SEC has largely been shown to overcome these issues, with EVs obtained in early fractions prior to the separation of soluble proteins and HDLs.^56^ In addition, respondents highlighted sample quality (36%), small sample sizes (41%) and time efficiency (44%) as factors governing the selection of SEC, all parameters advantageous to the study of biomarkers.^57,58^

PEG precipitation provides a simple, time efficient and scalable isolation method that has been previously applied for the isolation of virus particles.^59^ Outcomes from the present study identified PEG precipitation was solely applied by respondents working in a hospital setting (7% of total respondents). PEG precipitation was also applied where respondents did not have a primary focus on EV research (22% of total respondents). An increased use of PEG precipitation by those groups may be a reflection of the accessibility and relative ease of implementation in conjunction with ability to process both small- and large volume samples without specialist training or equipment.^28,60,61^ In addition, although there are some concerns surrounding the immunogenicity of PEG,^62,63^ the potential application of EVs isolated by PEG precipitation has been demonstrated clinically for the treatment of graft vs host disease^51^ and it is routinely applied in bio-pharmaceuticals.^64^ However, one major drawback of PEG precipitation is the co-isolation of proteins with EVs,^28,60,65^ which can impact specificity and reproducibility.^66^ This lack of specificity could be of particular significance if PEG precipitation is to be used diagnostically and suggests that there may be a compromise to be found between throughput, applicability and purity. However, positive outcomes have been observed when PEG is combined with washing steps and UC.^60^

There was no recorded use of SEC by respondents from an industry setting (only 2% of total respondents) or ***other*** research settings (9% of total respondents, research institutes and government), with SEC primarily utilised in academic settings (33%). For starting volumes >50 mL, we observed an increased use of SEC. When applying SEC to isolate from larger volumes, samples often need to be concentrated pre- and/or post-isolation.^39^ For example, the use of an ultrafiltration (UF) unit is frequently reported.^67–69^ However, careful consideration should be taken when selecting an UF unit, with different molecular weight cut-offs (MWCOs) and filter membranes found to alter the resulting EV preparations.^70^ Scalable approaches for sample concentration that are gaining traction within the field but not reported in the present study include SEC with TFF.^38,71,72^ Surprisingly, there was no recorded use of SEC when respondents had 10+ years EV experience (7% of total respondents). This is perhaps due to SEC having been more widely adopted in recent years. This aligns with the ISEV 2019 survey^32^ showing a significant increase, of approximately three times (2015 – 15% and 2019 – 45%) the number of respondents utilising SEC compared to 2015.^31^

Respondents in the early stages of their EV research (1–3 years EV experience, 57% of total respondents) applied a greater diversity of EV isolation methods. This included PEG precipitation and commercial reagents (1% and 17%, respectively), which were not utilised by those with over 3 years of experience. Respondents with over 3 years of experience had increased application of ***other*** methods (including TFF, DG, UF and MFs). An increase in ***other*** methods (23%) was also observed for respondents isolating EVs from urine, aligning with outcomes from the ISEV urine task force 2021 position paper.^53^ The application of methods such as TFF have been suggested to overcome scalability issues both at a manually operated small scale and fully automated GMP compliant manufacturing scale.^71,73–76^ The use of TFF was reported by three respondents (specified when selecting ***other*** methods) for applications in therapeutics and method development. An increasing application of TFF for EV isolation also aligns with ISEV surveys which had no recorded applications in 2014,^31^ in comparison to 12% in 2019.^32^ TFF has been shown to produce higher yield and purity outputs, as well as greater batch-to-batch consistency whilst exerting minimal force on the EVs.^74,77^ It is also an already established method for virus preparation and thus easily implementable for biomanufacturing.^78–80^ In addition, its clinical applicability has been previously demonstrated in trials for cancer immunotherapy,^72,81–83^ suggesting that the growing numbers of EV clinical studies may be one reason for its increased application. Although TFF has demonstrated potential, there are still limitations to overcome. For example, the use of protein rich media can lead to a reduction in purity.^77^ Therefore, TFF is often utilised in combination with SEC to enhance sample purity.^71,84^ This also true for the isolation of EVs from complex biofluids such as blood, due to the overlap in density of HDLs and EVs (1.063–1.21 g/mL and 1.13–1.19 g/mL, respectively).^85^ Further evidenced by the outcomes of this survey, where preferential application of a combination of methods (24% of total respondents) was observed when isolating EVs from blood (50%) and its components plasma (39%) and serum (38%). Additionally, a combination of methods was also favoured by respondents when considering sample quality (33%) and compatibility with downstream analysis (36%). However, sample purity can often come at the expense of bioactivity, with a number of studies indicating that increased purification can lead to the removal of biomolecular components necessary to elicit specific therapeutic responses.^86–89^ This suggests that the broader secretome or inclusion of a protein corona around EVs may be of therapeutic relevance.^90^ Consequently, this balance between purity and potency is perhaps one of the factors influencing the decreased use of a combination of methods in therapeutic applications (13%) and needs to be carefully evaluated when considering both therapeutic efficacy and regulatory requirements of developing EV therapeutics. We also observed a decreased application of a combination of methods with large sample volumes, likely due to lengthy multi-step processing where large-scale manufacturing (only 1% of total respondents) is not applied. This suggests that application may play a greater role in the selection of a combination of methods, with large sample volumes associated with therapeutic applications and small volumes for diagnostic applications. These outcomes further highlight how a balance between purity, efficacy and more pragmatic considerations will likely need to be established when working towards the development of EV diagnostics and therapeutics.

The aim of this survey was to evaluate factors governing the selection of more universally applied EV isolation methods. However, some respondents reported the use of ***other*** methods (10%, such as TFF, DG and UF), including the use of microfluidics (one respondent).^91,92^ This aligns with the previously mentioned ISEV surveys which had no recorded applications of microfluidics in 2014,^31^ in comparison to 4% in 2019.^32^ Microfluidics provides a rapid, accurate and potentially automatable method for processing minimal sample volumes.^93^ These systems also provide the opportunity to combine EV isolation with more in-depth biochemical and biophysical analysis,^94,95^ offering the potential to bridge the gap from bench to bedside, particularly for the high-throughput assessment of diagnostic biomarkers.^96,97^ Although, not specified by any respondents in this survey, the 2015 ISEV^31^ survey also indicated the use of magnetic bead separation techniques with both biological samples (13%) and small sample volumes (<1 mL, 28%),.^98,99^ Examples of immunoaffinity methods such as this and others reported in the literature (e.g. ferric oxide nanocubes^100^ and multiplexed gold sensors^101^) also have potential for the application of EVs as diagnostic biomarkers. With examples prevalent within the cancer field for the differentiation of chondroitin sulphate peptidoglycan 4 (CSPG4)^+^ melanoma tumour-derived vesicles from healthy controls^102^ and for the capture of epithelial cell adhesion molecule positive (EpCAM^+^) prostate EVs from prostate cancer cells.^103^ Lastly, other methods in the literature gaining interest for the isolation of EVs are those already established for biomanufacturing processes, such as the use of PEG and Dextran (DEX) to form an aqueous two-phase system (ATPS).^104,105^ ATPS has the potential to be a cost-effective isolation method that can obtain EVs from both small^106,107^ and large sample volumes^108^ without the need for specialist equipment and can reduce the co-isolation of proteins observed with other precipitation methods.^109^ However, final EV preparations are recovered in DEX, a highly viscous reagent which has been indicated to interfere with downstream analysis.^110^ In addition to a lack of understanding on the clinical safety and efficiency of DEX if to be applied therapeutically.

Overall, the outcomes of this survey highlighted the advantages and limitations of popular EV isolation methods based on parameters that govern their application and implementation. We anticipate these outcomes will aid researchers in their choice of method selection for EV isolation, in line with their individual research objectives. However, it should be noted that this study faces some limitations that require consideration. Firstly, most respondents surveyed were largely from an academic background (81%), with students and postdoctoral researchers accounting for the majority (79%) and academic staff accounting for 14%. Of these respondents, 93% of had reportedly worked in the field for less than 10 years. As such, outcomes of this study must be considered to communicate largely an overview of opinions from within the academic research community. Additionally, the technology readiness level (TRL) of each entry was not obtained. Based on the background of respondents, it is likely that commentary on the scalability of a given isolation method does not reflect the volumes required for downstream therapeutic EV manufacture but rather larger-scale pre-translational laboratory research. It is also possible that the large proportion of respondents who have been working with EVs for only a short period of time may have resulted in an increased selection of simple accessible one-step isolation protocols, as evidenced by the application of PEG and commercial precipitation reagents only by respondents with 1–3 years’ experience (Figure 4(b)). However, identifying these trends is important if we are to tackle potential issues with experimental reproducibility and address challenges with translation. Finally, efforts to survey a broader cohort of individuals from across industry and the clinical sciences will be important as the therapeutic translation of EVs gains traction.

## Summary and conclusion

Studies attempting to utilise EVs for diagnostics and therapeutic applications are increasing rapidly. This study reports the findings of our survey, providing a broad overview of up-to-date trends in utilisation of EV isolation methods and parameters that govern their selection. Enabling the very first cross-comparison study across disciplines and sectors to provide a comprehensive overview of the selection and application of EV isolation methods and the parameters governing their implementation. Results highlighted the diverse and specific nature of considerations when selecting an EV isolation method. These considerations take into account not only parameters such as EV source, starting volume and purity, but also more pragmatic considerations such as application, operator experience, research setting, throughput and implementation (e.g. cost and scalability). It is evident that in order to encourage reproducibility across the rapidly evolving EV field, awareness and open availability of the benefits and limitations of common EV isolation methods needs to be clearly and concisely communicated to individuals from a range of disciplines. It is also clear that the requirements of end users will vary considerably depending on the intended objective. Therefore, the EV community should aim to provide an open and accessible framework to guide those less familiar with standards in the field. This will aid clinical and commercial translation as the field continues to expand across disciplines.

## Supplemental Material

sj-docx-1-tej-10.1177_20417314231155114 – Supplemental material for A survey to evaluate parameters governing the selection and application of extracellular vesicle isolation methods Click here for additional data file. Supplemental material, sj-docx-1-tej-10.1177_20417314231155114 for A survey to evaluate parameters governing the selection and application of extracellular vesicle isolation methods by Soraya Williams, Aveen R Jalal, Mark P Lewis and Owen G Davies in Journal of Tissue Engineering
