# Supplementary material for: A survey to evaluate parameters governing the selection and application of extracellular vesicle isolation methods
Source: J Tissue Eng. 2023 Mar 8;14:20417314231155114. doi: 10.1177/20417314231155114 (PMC9996742; doi:10.1177/20417314231155114)
Supplement: sj-docx-1-tej-10.1177_20417314231155114 – Supplemental material for A survey to evaluate parameters governing the selection and application of extracellular vesicle isolation methods [file sj-docx-1-tej-10.1177_20417314231155114.docx]

Supplementary Information

# EV Researchers Experience of EV Isolation Methods Online Questionnaire

**EV Researchers Experience of EV Isolation Methods Online Questionnaire**

**PARTICIPANT INFORMATION**

**What is the purpose of the questionnaire?**A current limiting factor of EV research is the suboptimal EV isolation methods. My current PhD research compares five of the multitude of EV isolation methods, in order to understand their advantages and disadvantages for varied applications and research settings. The purpose of this questionnaire is to collate personal experience and opinions of EV isolation methods, from both new and established EV researchers, across the disciplines of EV research. The information collected will then be compared alongside my own findings, to give an up to date understanding of these applications and challenges of EV isolation methods within the wider EV community.

The survey consists of 11 short multiple-choice questions and should take around 4 minutes to complete.

**Are there any exclusion criteria?**
Your current research must involve Extracellular Vesicles  
You must have laboratory experience of isolating Extracellular Vesicles

**Investigators Details:**
 If you have any questions, please do not hesitate to contact the Principal Investigator, Soraya Williams *(s.williams3@lboro.ac.uk)*, or her PhD supervisor Dr Owen Davies *(O.G.Davies@lboro.ac.uk)* at Loughborough University, UK.

The questionnaire will complete once you have pressed the submit button on the final page.

Please answer the questions focusing on your own personal laboratory experiences.

**ONLINE SURVEY INFORMED CONSENT LANDING PAGE**

**Welcome to the EV Researchers Experience of EV Isolation Methods Survey**    

**Taking Part**
The purpose of this survey is to record your own personal experience and opinions of EV isolation methods.
This study is designed to further scientific knowledge and that all procedures have been approved by the Loughborough University Ethics Approvals (Human Participants) Sub-Committee.

 Please remember to answer the questions honestly throughout the survey.

You are under no obligation to take part in the study, you have the right to withdraw from this study at any stage for any reason and will not be required to explain your reasons for withdrawing.


**Use of Information**
I understand that all the personal information I provide will be processed in accordance with data protection legislation on the public task basis and will be treated in strict confidence unless (under the statutory obligations of the agencies which the researchers are working with), it is judged that confidentiality will have to be breached for the safety of the participant or others or for audit by regulatory authorities.


I understand that information I provide will be used for PhD research in the form of reports for research output purposes and scientific publication.

I agree that information I provide can be quoted anonymously in research outputs.

I agree to assign the copyright I hold in any materials related to this project to Soraya Williams.

I agree for the anonymised data I provide to be deposited in Qualtrics so that it can be made publicly available for future research at the end of the project.   


 I understand that cookies and personal data stored by your web browser are not used in this survey.

- **I voluntarily agree to take part in this study, for our records and to confirm your agreement with the above, please select**

Q1 What kind of  research setting do you work in?

- University
- Hospital
- Industry
- Other (please specify) ________________________________________________

Q2 What is your job title?

- Undergraduate Student
- Masters Student
- PhD Student
- Postdoctoral Researcher
- Academic
- Industry Scientist
- Clinical Research Scientist
- Clinician
- Other (please specify) ________________________________________________

Q3 How long have your worked with EVs?

- 1-3 years
- 4-9 years
- 10+ years

Q4 Is EV research your primary area of focus?

- Yes
- No

Q5 What fluid source do you isolate EVs from, ***select ALL that apply***

- Cell Culture Media
- Urine
- Blood
- Plasma
- Serum
- Saliva
- Other (please specify) ________________________________________________

Q6 What is the ***MAIN***focus of your EV work?

- Therapeutics
- Diagnostics
- Method Development
- Characterisation
- Manufacturing
- Regulation
- Other (please specify) ________________________________________________

Q7   What volume do you typically isolate from? 
 *(Volume selected should be per individual sample before pooling)*

- Up to and including 2ml
- Between 2ml - 50ml
- More than 50ml

Q8 Are you currently able to isolate enough EVs for your intended outcome?

- Yes
- No

Q9 What is your current ***MAIN*** method of EV isolation?

- Ultracentrifugation
- PEG Precipitation
- Commercial Reagent *(e.g. Total Exosome Isolation Reagent)*
- Aqueous Two-Phase System
- Size Exclusion Chromatography
- Combination of Methods
- Other (please specify) ________________________________________________

Q10 Rank each aspect of these EV isolation methods based on your

***OWN PERSONAL LABORATORY EXPERIENCE***

*This question is being utilised in another study*

Q11  What are the main factors that influenced you to choose your current method, ***select ALL that apply:***

- Cost
- Equipment accessibility
- Time efficiency
- EV output
- Sample quality
- Ability to process small sample sizes
- Ability to process large sample volumes
- Analysis method(s)
- Method routinely used within my group/lab
- Limited experience/knowledge of other methods
- Other (Please Specify) ________________________________________________
